# Outcomes from patients with presumed drug resistant tuberculosis in five reference centers in Brazil

**DOI:** 10.1186/s12879-017-2669-1

**Published:** 2017-08-15

**Authors:** D. M. P. Ramalho, P. F. C. Miranda, M. K. Andrade, T. Brígido, M. P. Dalcolmo, E. Mesquita, C. F. Dias, A. N. Gambirasio, J. Ueleres Braga, A. Detjen, P. P. J. Phillips, I. Langley, P. I. Fujiwara, S. B. Squire, M. M. Oliveira, A. L. Kritski, for Rede-TB Study group

**Affiliations:** 1 Tuberculosis Academic Program, Medical School and Hospital Complex HUCFF-IDT, Federal University of Rio de Janeiro, Rio de Janeiro, Brazil; 2 Helio Fraga Reference Center – ENSP –Fiocruz, Rio de Janeiro, Brazil; 3 Messejana Hospital –State Secretary of Health, Fortaleza, Ceará Brazil; 4 Ary Parreiras Institute – State Secretary of Health, Rio de Janeiro, Brazil; 5 Sanatório Partenon Hospital - State Secretary of Health, Porto Alegre, Rio Grande do Sul Brazil; 6 Clemente Ferreira Institute - State Secretary of Health, Sao Paulo, Brazil; 7 International Union Against Tuberculosis and Lung Disease, Paris, France; 8 Liverpool School of Tropical Medicine, Liverpool, UK; 9 MRC Clinical Trials Unit, London, UK

**Keywords:** Multi-drug resistant tuberculosis, Diagnosis, Treatment outcome

## Abstract

**Background:**

The implementation of rapid drug susceptibility testing (DST) is a current global priority for TB control. However, data are scarce on patient-relevant outcomes for presumptive diagnosis of drug-resistant tuberculosis (pDR-TB) evaluated under field conditions in high burden countries.

**Methods:**

Observational study of pDR-TB patients referred by primary and secondary health units. TB reference centers addressing DR-TB in five cities in Brazil. Patients age 18 years and older were eligible if pDR-TB, culture positive results for *Mycobacterium tuberculosis* and, if no prior DST results from another laboratory were used by a physician to start anti-TB treatment. The outcome measures were median time from triage to initiating appropriate anti-TB treatment, empirical treatment and, the treatment outcomes.

**Results:**

Between February,16th, 2011 and February, 15th, 2012, among 175 pDR TB cases, 110 (63.0%) confirmed TB cases with DST results were enrolled. Among study participants, 72 (65.5%) were male and 62 (56.4%) aged 26 to 45 years. At triage, empirical treatment was given to 106 (96.0%) subjects. Among those, 85 were treated with first line drugs and 21 with second line. Median time for DST results was 69.5 [interquartile - IQR: 35.7–111.0] days and, for initiating appropriate anti-TB treatment, the median time was 1.0 (IQR: 0–41.2) days. Among 95 patients that were followed-up during the first 6 month period, 24 (25.3%; IC: 17.5%–34.9%) changed or initiated the treatment after DST results: 16/29 MDRTB, 5/21 DR-TB and 3/45 DS-TB cases. Comparing the treatment outcome to DS-TB cases, MDRTB had higher proportions changing or initiating treatment after DST results (*p* = 0.01) and favorable outcomes (*p* = 0.07).

**Conclusions:**

This study shows a high rate of empirical treatment and long delay for DST results. Strategies to speed up the detection and early treatment of drug resistant TB should be prioritized.

## Background

In 2015, WHO estimates that there were about 580,000 newly eligible for multidrug resistant tuberculosis (MDR-TB) treatment. Nevertheless, only 125,000 (20%) were enrolled. Approximately 60% of them occurred in Brazil, China, India, the Russian Federation, South Africa, Indonesia and Nigeria [1, 2]. Low rates of treatment completion or cure (58 to 67%) in MDR-TB have been described in a recent systematic reviews and meta-analyses [3–6]. In order to respond more effectively to the emergence of co-infection with TB and HIV and MDR-TB globally, WHO has recommended new TB diagnostic technologies, and most recently, rapid drug susceptibility testing using molecular Line Probe Assays or Xpert MTB RIF [7, 8]. The implementation of these techniques should help programs to cope with the current clinical management demands and also may help implementation of the new anti-TB regimens that are in the pipeline.

However, only a handful studies of the clinical impact on adult patients with a presumed diagnosis of drug-resistant tuberculosis (DR-TB) under field conditions in high burden countries have been published regarding the incorporation of new molecular technologies for TB diagnosis.

Clinicians often start empirical TB treatment regimens before culture and DST results become available. There are several reasons for this, including the fact that culture and DST results often take a long time and, sometimes, do not even become available at all. This tradition of empirical treatment has major implications on the impact of newer and more sensitive diagnostic tests with faster laboratory turnaround times [9–19].

In Brazil, much advancement in tuberculosis control in the past 10 years have been described. Despite all this progress, some very serious obstacles still need to be addressed, including the low rate of detection of drug-resistant TB and, also, the high morbidity and mortality rate among MDR-TB cases [20]. In 2012, culture was performed in 17.4% of TB cases reported. Only 28.1% of those cases were previously treated TB cases. MTBDRplus, Xpert™ MTB/Rif and MGIT960 have been commercialized in Brazil, even though without being formally incorporated into the public health system for the diagnosis of DR/MDR-TB. Still, no data is available regarding the use of these new diagnostic technologies in public TB reference centers that manage DR/MDR-TB. In 2015, plans were made to implement Xpert™ MTB/Rif in 90 municipalities, covering 55% of the TB burden in the country. According to this, it was expected that the detection of DR-TB cases would increase 3 to 4 fold [21]. In order to assist the impact evaluation of the incorporation of these new molecular tests in the Brazilian Unified Health System (SUS), the International Union Against Tuberculosis and Lung Disease (The Union), through the TREAT TB initiative, the Brazilian Network of Tuberculosis Research (REDE-TB) and, the Academic Tuberculosis Program of the Federal University of Rio de Janeiro, proposed a crossover randomized pragmatic clinical trial (Register N. RBR-4rprbd). Prior to this trial an observational and descriptive baseline study among pDR-TB cases evaluated in State Reference Centers has been completed and is reported here. The objectives of this observational study were to describe the clinical and laboratory management of patients with pDR-TB and to describe risk factors for DR/MDR-TB in patients attending DR-TB reference centers in four Brazilian states.

## Methods

### Setting

Five sites from four different regions in Brazil were included: inpatient services at Hospital Sanatorio Partenon-Secretaria Estadual de Saúde do Rio Grande do Sul (HU-SES-RGS), Hospital Messejana – Secretaria Estadual do Ceará (HM-SES-CE), Instituto Estadual Ary Parreiras – Secretaria Estadual do Rio de Janeiro (IETAP-SES-RJ), outpatient services at Instituto Clemente Ferreira – Secretaria Estadual de São Paulo (ICF-SES-SP) and, Centro de Referência Hélio Fraga – Fundação Oswaldo Cruz – Rio de Janeiro (CRPHF-Fiocruz-RJ).

### Participants

Eligible participants were any patients aged 18 years and older with cough for 3 weeks or more and, in accordance with national guidance [22], with the presence of at least one of the following social-clinical conditions defining them as pDR TB at triage as follows: (a) suspected re-treatment failure or previous treatment default; (b) HIV seropositive subjects, c) close contact with smear positive MDR-TB cases, (d) homeless or e) hospitalization in TB reference centers. All participants gave written Informed Consent. Subjects were excluded if they: (a) had DST results (drug resistant or drug sensitive) from another laboratory that had been used by an attending physician to start anti-TB treatment at triage; (b) had no laboratory assessment of the drug sensitivity confirmation of M. tuberculosis; (c) were harboring environmental mycobacteria and, (d) had no clinical and/or laboratory results available in the medical records.

### Data collection

Local study staff was comprised by one of each of the following: a attending physician, a nurse, and a laboratory technician that belonged to the professional staff of the Health Unit. During the study period, the survey was carried out by the study staff on all presumed drug resistant TB that fulfilled the eligibility criteria and was attended consecutively in five Health Units. Prospectively, routinely collected clinical data were extracted onto a Study Form from patient registers and clinical records. Patient registers contained information on all included patients listed in consecutive order with name, age, sex, address, phone number, type of patients (previous treatment classification) and date of diagnosis (when available). Also, it was collected from medical records, clinical data, socio-demographics, previous treatment failures or defaults, HIV status and hospitalization. Clinical samples collected in Health Units were sent to a local laboratory using standard practice. Laboratories issued results according to routine procedures. All clinical samples from these 5 sites were sent to the local Mycobacterial Laboratory for smear microscopy, culture, drug susceptibility testing and identification at species level. Participants were assigned to have their samples submitted to the following routine bacteriological tests: BACTEC™ MGIT 960™ Mycobacterial Detection System (MGIT960) or Lowenstein-Jensen (LJ)/Ogawa Kudo (OK) and Proportion Method (PM), according to the Brazilian Tuberculosis National Guidelines [22]. Tests were performed according to laboratory routine and the techniques used are all fully described [23]. Clinical and radiological improvement was judged by the attending physicians at each site. TB patients received anti-TB treatment and clinical follow-up from the attending physician as routinely planned in the local algorithm. In summary, routine clinical procedures were not affected by this study. Every 2 months, data monitoring research team checked all clinical and laboratory data collected.

### Case definition

Drug resistant (DR-TB) cases were defined as those harboring *M. tb* isolates resistant to one or more drugs and multidrug resistant (MDR-TB) cases as resistant to at least Rifampin-RIF and Isoniazid-INH. Drug sensitive (DS-TB) cases were defined as those harboring *M.tb* isolates susceptible to all first-line anti-TB drugs (RMP, INH, Ethambutol-EMB, Pyrazinamid-PZA and Streptomycin-SM). Empirical treatment was defined when at triage physicians started TB treatment regimen before DST results were available. Appropriate anti-TB treatment was defined accordingly to the regimen prescribed by the attending physician (matching DST results). Interim treatment outcomes were evaluated after 6 months of enrollment using available data from medical records. Retreatment cases were grouped according to the outcome of previous treatment: cured, completed, defaulted or failed. A patient was defined as cured when tested smear-negative at treatment completion or, at least in one of the previous test.

A completed treatment was defined as patients who completed treatment but without smear microscopy proof of cure. Persons who had treatment interruption for two consecutive months or more were grouped as defaulted. Those who remained smear-positive when tested five or 6 months after initiation of their previous treatment were defined as treatment failures. Clinical or radiological improvement and/or culture conversion were considered favorable TB treatment responses. Death from any cause and default were unfavorable. Transferred care cases which had no outcome data were all excluded.

### Endpoints

A standard form was created in order to collect data regarding to the following timing: triage (screening visit), sputum collection, DST result released by laboratory, DST results seen by the physician and, initiation or change of TB treatment regimen after DST results. The primary endpoint was time from triage to initiation or change of TB treatment regimen because of the DST result. The secondary endpoint was the proportion of favorable treatment outcomes according to the initial empirical treatment regimen prescribed as follows: with first line drug regimen (RIF + INH + EMB + PZA) or with second line drug regimen (ethionamide, levofloxacin, amikacyn/capreomicyin, clofazimine).

### Statistical analysis

We compared socio-demographic and clinical characteristics between included and excluded patients. Also, we identified the factors associated with drug resistance. Exploratory analysis was carried out through dichotomous outcomes based on the calculation proportion for all the different groups. For continuous outcomes, median and interquartiles were used. Sample distribution of time periods from triage to DST results and to initiate or change TB treatment were compared. Fisher’s Exact Test with mid-p correction for comparisons between proportions was used, as well as the Mann-Whitney Test to compare differences in morbidity. All analyses were performed using SPSS software (version 17).

The protocol was approved by the National Research Ethics Committee (CONEP **N°** 520/2011; Register: 16,571 – Process: n° 25,000.115789/2011–94) and by the Ethics Advisory Group at The Union, number: 11/11. The protocol was also approved by each appropriate local Institutional Review Board and Ethics Committee.

## Results

**Fig. 1:**
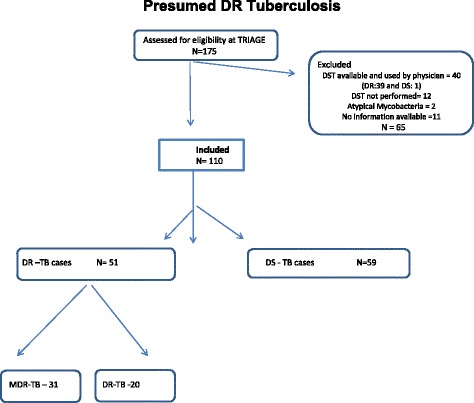
Flow diagram for Baseline Prove IT/5 sites 2011–2012. Amongst the 175 pacientes, 110 were included in the study, being 51(46%) drug resistant and. Of these, 31(61%) are multi drug resistant.

A total of 175 eligible presumed DR-TB patients were evaluated and 110 (63.0%) enrolled. Among the 65 excluded cases, the physicians used previous DST for decision making in 40 (DR: 39 and DS:1); 12 cases had no DST results available, 11 cases had no additional clinical and/or laboratory results available at the medical records, and two had growing atypical mycobacteria (Fig. 1- Flow diagram for Baseline Prove IT/5 sites 2011–2012). A higher proportion of exclusion was observed at CRPHF-Fiocruz-RJ (55.0%; 22/40) and lower at HP-SES-RGS (14.0%; 6/43) (data not shown). The 65 excluded patients were similar to the 110 included in all socio-demographic, clinical and behavioral respects, except for higher schooling status (Table 1: Comparison of sociodemographic, behavior and clinical characteristics of presumed DR TB cases included and excluded in the study).

**Table 1:** Comparison of sociodemographic, behavior and clinical characteristics of Presumed DR TB cases included and excluded in the study

Variable	Presumed DR TB cases		*P* value
	Included *N* = 110 n (%)	Excluded *N* = 65 n (%)	
Demographic characteristics			
Sex			
Male	72(65.5)	44(67.7)	*p* = 0.76
Female	38(34.5)	21(32.3)	
Age			
< 25	15(13.6)	12(18.5)	
26–45	62(56.4)	34(52.3)	*p* = 0.39
> 45	33(30.0)	19(29.2)	*p* = 0.49
Marital status			
Single	78(70.9)	49(75.4)	
Married	32(29.1)	16(24.6)	*p* = 0.52
Race			
White	46(41.8)	18(27.7)	*p* = 0.06
Non White	64(58.2)	47(72.3)	
Schooling			
< 8 years	98(89.1)	47(72.3)	
> 8 years	9(8.2)	14(21.5)	*p* = 0.08
IGN	3(2.7)	4(6.2)	*p* = 0.17
Behaviour Characteristics			
Smoker			
Current/Ex	83(75.5)	45(69.2)	*p* = 0.34
Never	25(22.7)	19(29.2)	
IGN	2(1.8)	1(1.5)	
Alcoholism (CAGE)			
Yes	20(18.2)	13(20.0)	*p* = 0.76
No	90(81.8)	52(80.0)	
Admission hospital last 2 years			
Yes	37(33.6)	26(40.0)	*p* = 0.42
No	72(65.5)	39(60.0)	
IGN	1(0.9)	0	
Admission prision last 2 years			
Yes	21(19.1)	14(21.5)	*p* = 0.65
No	89(80.9)	50(76.9)	
IGN	0	1(1.5)	
Admission in Shelters last 2 years			
Yes	8(7.3)	10(15.4)	*p* = 0.09
No	100(90.9)	55(84.6)	
IGN	2(1.8)	0	
Clinical, Radiological and Laboratory Characteristics			
HIV testing result			
Positive	12(10.9)	13(20.0)	*p* = 0.26
Negative	61(55.5)	40(61.5)	
IGN	37(33.6)	12(18.5)	
Contact of pulm TB			
Yes	55(50.0)	32(49.2)	*p* = 0.52
No	45(40.9)	21(32.3)	
IGN	10(9.1)	12(18.5)	
TB in the past			
Yes	87(79.1)	47(72.3)	*p* = 0.42
No	22(20.0)	16(24.6)	
IGN	1(0.9)	2(3.1)	
Number treat in the past			
= > 3	31(28.2)	13(20.0)	*p* = 0.34
< 3	56(50.9)	34(52.3)	
IGN	23(20.9)	18(27.7)	
Form TB			
Pulmonary	85(77.3)	46(70.8)	*p* = 1.00
Pulmonary + Extrapulmonary	2(1.8)	1(1.5)	
NA	23(20.9)	18(27.7)	
Weight loss			
Yes	75(68.2)	43(66.2)	*p* = 0.78
No	35(31.8)	22(33.8)	
Cough			
Yes	101(91.8)	62(95.4)	*p* = 0.88
No	7(6.4)	3(4.6)	
IGN	2(1.8)	0	
Expectoration			
Yes	95(86.4)	53(81.5)	*p* = 0.31
No	14(12.7)	12(18.5)	
IGN	1(0.9)	0	
Hemoptysis			
Yes	26(23.6)	14(21.5)	*p* = 0.76
No	83(75.5)	50(76.9)	
IGN	1(0.9)	1(1.5)	
Sweating			
Yes	68(61.8)	38(58.5)	*p* = 0.32
No	35(31.8)	27(41.5)	
IGN	7(6.4)	0	
Fever			
Yes	67(60.9)	38(58.5)	*p* = 0.61
No	39(35.5)	26(40.0)	
IGN	4(3.6)	1(1.5)	
Loss apetite			
Yes	72(65.5)	47(72.3)	*p* = 0.43
No	36(32.7)	18(27.7)	
IGN	2(1.8)	0	
Chest wheezing			
Yes	57(51.8)	33(50.8)	*p* = 0.63
No	46(41.8)	31(47.7)	
IGN	7(6.4)	1(1.5)	
Dyspneia			
Yes	74(67.3)	35(53.8)	*p* = 0.06
No	34(30.9)	30(46.2)	
IGN	2(1.8)	0	
Sneezing			
Yes	45(40.9)	24(36.9)	*p* = 0.41
No	59(53.6)	41(63.1)	
IGN	6(5.5)	0	

*IGN* ignored, *NA* not applicable

Among those included were: 72 (65.5%) males, 62 (56.4%) aged 26 to 45 years, 20 (18.2%) with alcoholism, 83 (75.5%) current/ex-smokers, 37 (33.6%) admitted in hospitals, 21 (19.0%) referred as staying in prison during the last 2 years and, 3 (2.7%) type 2 diabetes mellitus. HIV infection was identified in 12 (10.9%) and contact with TB was shown in 50 (55.0%). TB in the past was identified in 87 (79.1%), and among them, 31 (28.2%) with more than three treatments (Table 1).

Time (in days) from triage to sputum collection was 0.0 (interquartile-IQR: 0–1.0); from triage to culture results was 32.5 (IQR: 14.7–63.0); from triage to DST results released by laboratory was 69.5 (IQR: 35.7–111.0); from triage to DST results seen by the physicians was 97.0 (IQR: 64.2–143.0) and, from triage to adoption of appropriate TB treatment, it was 1.0 (IQR: 0–41.2) [Table 2 – Description of time from triage and different medical and laboratory procedures among 110 presumed DR TB suspects]. The median time (in days) from triage to DST results released by the laboratory [133.0 (IQR: 95.0–184.0)] and, time from triage to DST results seen by physicians [139.0 (IQR: 96.0–185.0)], were the longest at IETAP-SES-RJ. The time (in days) from triage to DST results released by laboratory [35.0 (IQR: 27.0–58.7)] and time from triage to DST results seen by the physicians, [65.0 (IQR:36.0–86.0)] were the shortest at HM-SES-CE. The median time (in days) from triage to appropriate TB treatment was the longest at ICF-SES-SP [35 (IQR: 35.0–99.0)], and the shortest at HP-SES-RS [0 (IQR: 0–5.0)] (Table 2).

**Table 2:** Description of time from Triage and different medical and laboratorial procedures among 110 presumed DR TB suspects

Sites (type of culture used)		To sputum collection	To treatment onset	To culture results	to DST results released by Lab	to DST results seen by physicians	To appropriate treatment
Messejana hospital	N	32	32	32	32	31	32
(culture and DST by MGIT)	Median	0	0	16.5	35	65	31
	IQR	0–0	0–10.7	10–41,2	27–58.7	36–86	0–55
Clemente Ferreira Institute	N	19	19	19	19	17	19
(culture and DST by MGIT)	Median	0	0	11	59	128	35
	IQR	0–1	0–0	9–18	26–115	57–159	35–99
Ary Parreiras hospital	N	15	14	15	15	15	15
(culture and DST by MGIT = 14)	Median	1	0	133	133	139	1
(culture and DST by PM = 1)	IQR	0–3	0–1	89–180	95–184	96–185	0–77
Helio Fraga Reference Center	N	17	17	17	13	9	17
(culture and DST by MGIT = 16)	Median	0	0	43	87	141	1
(culture and DST by PM = 1)	IQR	0–0	0–5	30.5–54.5	79–115.5	100–151	0–67.5
Partenon Hospital	N	27	27	27	27	24	27
(culture and DST by MGIT = 11)	Median	0	0	40	70	100.5	0
(culture and DST by PM = 16)	IQR	0–1	0–1	30–63	63–113	70.2–149.7	0–5
All sites	N	110	109	110	106	96	110
(culture and DST by MGIT = 92)	Median	0	0	32.5	69.5	97	1
(culture and DST by PM = 18)	IQR	0–1	0–1	14.7–63	35.7–111	64.2–143	0–41.2

*DST* drug susceptibility testing, *IQR* interquartile

Culture results were provided by solid medium in 55 (45.1%) cases [LJ:2 (1.6%), Ogawa Kudo: 53 (43.4%)] and MGIT960 in 67 (54.9%) cases. Smear positive/culture positive and smear negative/culture positive cases were identified in 96 (84.2%) and 16 (14.0%) cases, respectively. Two cases did not have smears but had positive cultures. DST results were available on 110 cases: 18 (16.4%) performed by the PM and, 92 (83.6%) where the MGIT960 was used. DST results were provided by PM more frequently in HSP-SES-RS. The median time (in days) of laboratory released results of susceptibility testing was similar with solid medium 70 (IQR: 63.0–113.5) as that observed with liquid medium 67 (IQR:33.5–114.5) [data not shown].

The distribution of drug resistance is shown in Table 3 (Resistance for 1st line anti-TB drugs among *M.tuberculosis* isolates from presumed drug resistant TB cases en 5 Reference Centers – Brazil). Overall, DR and MDR-TB rates were high: 51 (46.4%, 95% CI 37.3–55.6) and, 28 (25.5%, 95% CI 18.2–34.4) cases, respectively. Among subjects not previously treated, 22 had MDR (25.3%, 95% CI 17.3%–35.4%). Among previously treated subjects, 6 had MDR (26.1%, 95% CI 12.3–46.7). Resistance rates were particularly high for INH (*n* = 35, 40.2%, RIF *n* = 24, 27.6% and EMB (*n* = 16, 18.4%). Among the 24 *M tuberculosis* strains resistant to RMP, 23 (96.0%; 95% CI: 78.0–99.0) were also resistant to INH.

**Table 3:** Resistance for 1st line anti-TB drugs among *M.tuberculos*is isolates from presumed Drug Resistant TB cases in 5 Reference Centers - Brazil

Resistance	Isolates from New cases (*n* = 87)		Isolates from retreatment cases (*n* = 23)	
	N	% (95% CI)	N	% (95% CI)
Fully susceptible	48	55.2(44.7–65.2)	11	50.0(30.7–69.3)
Any resistance^a^				
INH	35	40.2(30.5–50.7)	10	45.4(26.9–63.4)
RMP	24	27.6(19.3–37.8)	7	31.8(16.2–52.8)
EMB	16	18.4(11.5–27.9)	1	4.5(0.1–29.0)
SM	14	16.1(9.7–25.3)	4	18.9(6.7–39.1)
PZA	6	6.9(2.9–14.5)	1	4.5(0.1–29.0)
Multidrug resistance	22	25.3(17.3–35.4)	6	27.3(12.9–48.4)
RMP + INH	7	8.1(3.7–15.5)	3	13.6(3.9–34.2)
RMP + INH + EMB	2	2.3(0.1–6.7)	−	−
RMP + INH + SM	1	1.2(0.05–6.7)	2	9.1(1.3–29.0)
RMP + INH + EMB + SM	7	8.1(3.7–15.5)	−	−
Other forms of Resistance	5	5.7(2.2–13.1)	1	4.5(0.1–29.0)
INH only	6	6.9(2.9–14.5)	2	9.1(1.3–29.0)
RMP only	1	1.2(0.05–6.7)	−	−
SM only	1	1.2(0.05–6.7)	1	4.5(0.1–29.0)
EMB only	1	1.2(0.05–6.7)	−	−
PZA only	1	1.2(0.05–6.7)	−	−
Number of drugs to which patients were resistant				
0	48	55.2(44.7–65.2)	11	50.0(30.7–69.3)
1	10	11.5(6.2–20.1)	3	13.6(3.9–34.2)
2	13	14.9(8.8–24.0)	4	18.9(6.7–39.1)
3	5	5.7(2.2–13.1)	4	18.9(6.7–39.1)
4	11	12.6(7.0–21.4)	1	50.0(30.7–69.3)

*RMP* rifampicin, *INH* isoniazid, *EMB* ethambutol, *SM* streptomycin, *PZA* pyrazinamid, *CI* confidence intervals

^a^Any resistance indicates resistance to the anti-tuberculosis medication tested, independent of resistance results to other medications

Among 110 pDR-TB evaluated at triage, empirical treatment was identified in 106 (96.0%). Arriving at triage, 36 (33.0%) patients referred by primary or secondary health units were already taking anti-TB treatment, including 34 using standardized first line regimen and two with second line drugs. At triage, TB drugs were prescribed by the attending specialist as follows: 70 with first-line regimen and 21 with second line drugs.

Comparing the demographics, behavior and clinical characteristics of DR/MDR-TB to DS-TB cases and, MDR-TB to DS-TB cases, each one, separately, presented the following results: lower proportions of co-morbidity (*p* = 0.05; *p* = 0.04); weight loss (*p* < 0.01; *p* < 0.01); sweating (*p* = 0.04; *p* = 0.01) and, dyspnea (*p* = 0.05; *p* = 0.02) [Table 4 – Demographic, behavior and clinical characteristics of presumed drug resistant TB at triage].

**Table 4:** Demographic, Behaviour and Clinical Characteristics of Presumed Drug Resistant TB at Triage

	DS-TB	DR/MDR-TB			MDR-TB		*p value*
	N (%)	N (%)	OR(95% CI)	*p value*	N (%)	OR(95% CI)	
Demographic Characteristics							
*S*ex							
Male	39(66.1)	33(64.7)	1.0 (Reference)	*p* = 0.87	20(64.5)	1.0 (Reference)	*p* = 0.88
Female	20(33.9)	18(35.3)	0.94(0.42–2.06)		11(35.5)	1.07(0.43–2.67)	
Age							
< 25	9(15.3)	6(11.8)	1.0 (Reference)		4(12.9)	1.0 (Reference)	
26–45	34(57.6)	28(54.9)	0.81(0.25–2.55)	*p* = 0,71	18(58.1)	0.83(0.22–3.10)	*p* = 0.79
> 45	16(27.1)	17(33.3)	0.62(0.18–2.16)	*p* = 0.46	9(29.0)	0.79(0.18–3.31)	*p* = 0.74
Race							
White	20(33.9)	26(50.9)	1.0 (Reference)	*p* = 0.07	16(51.6)	1.0 (Reference)	*p* = 0.10
Non White	39(63.1)	25(49.1)	2.02(0.94–4.37)		15(48.4)	2.08(0.85–5.05)	
Schooling (Patient)							
< 8 years	54(91.5)	44(86.3)	1.0 (Reference)	*p* = 0.54	27(87.1)	1.0 (Reference)	*p* = 0.61
> 8 years	4(6.8)	5(9.8)	0.65(0.16–2.57)		3(9.7)	2.66(0.14–3.20)	
IGN	1(1.7)	2(3.9)	-		1(3.2)		
Behavhiour characteristics							
Smoker							
Current	30(50.8)	18(35.3)	1.0 (Reference)		10(32.3)	1.0 (Reference)	*p* = 0.10
Ex	18(30.5)	17(33.3)	0.63(0.26–1.53)	*p* = 0.31	11(35.4)	0.54(0.19–1.53)	*p* = 0.25
Never	10(16.8)	15(29.4)	0.40(0.14–1.07)	*p* = 0.06	10(32.3)	0.33(0.10–1.03)	
IGN	1(1.7)	1(2.0)			0		
Alcoholism (CAGE)							
Yes	13(22.0)	7(13.7)	1.0 (Reference)	*p* = 0.26	3(9.7)	1.0 (Reference)	
No	46(78.0)	44(86.3)	0.56(0.20–1.54) -		28(90.3	0.37(0.09–1.44)	*p* = 0.05
Admission hospital last 2 years							
Yes	22(37.3)	15(29.4)	1.0 (Reference)	*p* = 0.34	8(25.8)	1.0 (Reference)	*p* = 0.24
No	36(61.0)	36(70.6)	0.68(0.30–1.52)		23(74.2)	0.56(0.21–1.49)	
IGN	1(1.7)	0	-		0		
Admission prison last 2 years							
Yes	14(23.7)	7(13.7)	1.0 (Reference)	*p* = 0.18	5(16.1)	1.0 (Reference)	*p* = 0.40
No	45(76.3)	44(86.3)	0.51(0.18–1.38) -		26(83.9)	0.61(0.19–1.91)	
Admission in Shelters last 2 years							
Yes	5(8.5)	3(5.9)	1.0 (Reference)	*p* = 0.60	3(9.7)	1.0 (Reference)	*p* = 1.00
No	53(89.8)	47(92.1)	0.67(0.15–2.98)		27(87.1)	1.17(0.26–5.30)	
IGN	1(1.7)	1(2.0)	-		1(3.2)		
Clinical Characteristics							
Contact of pulm TB							
Yes	29(49.2)	26(50.9)	1.0 (Reference)	*p* = 0.95	19(61.3)	1.0 (Reference)	*p* = 0.17
No	24(40.7)	21(41.2)	1.02(0.46–2.25)		8(25;8)	1.96(0.73–5.27)	
IGN	6(10.1)	4(7.9)	-		4(12.9)		
TB in the past							
Yes	48(81.3)	39(76.5)	1.0 (Reference)	*p* = 0.66	23(74.2)	1.0 (Reference)	*p* = 0.60
No	11(18.7)	11(21.6)	0.81(0.31–2.07)		7(22.6)	0.75(0.25–2.19)	
IGN	0	1(1.9)	-		1(3.2)		
Number treat in the past							
= > 3	16(27.1)	15(29.4)	1.0 (Reference)	*p* = 0.62	9(29.0)	1.0 (Reference)	*p* = 0.63
< 3	32(54.2)	24(47.1)	1.25(0.51–3.01)		14(45.2)	1.28(0.45–3.60)	
IGN	11(18.7)	12(23.5)	-		8(25.8)		
Outcome TB treat in the past							
Cure/complete	17(28.8)	8 (15.7)	1.0 (Reference)	*p* = 0.17	3(9.6)	1.0 (Reference)	*p* = 0.06
Defaulting/failure	30(50.8)	28(54.9)	0.5(0.18–1.35)		19(61.3)	0.27 (0.07–1.08)	
IGN	12(20.3)	15(29.4)			9(29.1)		
Cough/Expectoration							
Yes	52(88.1)	43(84.3)	1.0 (Reference)	*p* = 0.40	23(74.3)	1.0 (Reference)	*p* = 0.06
No	6(10.2)	8(15.7)	0.62(0.19–1.92)		8(25;8)	0.33(0.10–1.06)	
IGN	1(1.7)	0	-		0		
Hemoptysis							
Yes	13(22.0)	13(25.5)	1.0 (Reference)	*p* = 0.70	6(19.3)	1.0 (Reference)	*p* = 0.73
No	45(76.3)	38(74.5)	1.18(0.49–2.86)		25(80.7)	0.83(0.28–2.45)	
IGN	1(1.7)	0	-		0		
Fever							
Yes	38(64.4)	29(56.8)	1.0 (Reference)	*p* = 0.29	17(54.8)	1.0 (Reference)	*p* = 0.22
No	18(30.5)	21(41.2)	0.65(0.29–1.44)		14(45.2)	0.57(0.23–1.41)	
IGN	3(5.1)	1(2.0)	-		0		
Weight loss							
Yes	49(83.1)	26(50.9)	4.71(1.96–11.3)	*p* < 0.01	14(45.2)	1.0 (Reference)	*p* < 0.01
No	10(16.9)	25(49.1)	1.0 (Reference)		17(54.8)	5.95(2.23–15.87)	
Sweating							
Yes	43(72.9)	25(49.0)	2.58(1.12–5.95)	*p* = 0.04	13(41.9)	1.0 (Reference)	*p* = 0.01
No	14(23.6)	21(41.2)	1.0 (Reference)		15(48.4)	3.54(1.36–9.22)	
IGN	2(2.5)	5(9.8)	-		3(9.7)		
Dyspneia							
Yes	44(74.6)	27(52.9)	2.39(1.05–5.44)	*p* = 0.05	15(48.4)	1.0 (Reference)	*p* = 0.02
No	15(25.4)	22(43.1)	1.0 (Reference)		16(51.6)	3.18(1.28–7.93)	
IGN	0	2(4.0)			0		

*IGN* ignored, *NA* not applicable, *MDR* multidrugresistant, *DR* drug resistant, *DS* drug sensitive

Comparing the radiological and Laboratory Results and Treatment Prescription at Triage to DS-TB cases, MDR TB cases were associated with lower proportion of typical image on chest x ray (*p* = 0.03) [Table 5 – Radiological and laboratory results and treatment prescription at triage and during the follow-up in 110 presumed drug resistant tuberculosis cases].

**Table 5:** Radiological and Laboratory Results and Treatment Prescription at Triage and during the follow-up in 110 Presumed Drug Resistant Tuberculosis Cases

Variable	DS-TB	DR/MDR-TB			MDR-TB		*p value*
	N (%)	N (%)	OR(95% CI)	*p value*	N (%)	OR(95% CI)	
Treatment and Laboratory results at Triage							
AFB at triagem							
Pos	53(89.8)	41(80.4)	1.0 (Reference)	*p* = 0.34	24(77.4)	1.0 (Reference)	*p* = 0.19
Neg	6(10.2)	8(15.7)	0.58(0.18–1.80)		6(19.3)	0.45 (0.13–1.55)	
IGN	0	2(3.9)			1(3.2)		
HIV testing result							
Positive	9(15.3)	3(5.9)	1.0 (Reference)	*p* = 0.06	3(9.7)	1.0 (Reference)	*p* = 0.33
Negative	27(45.8)	34(62.7)	0.26(0.06–1.07)		18(56.1)	0.50 (0.11–2.10)	
IGN	11(38.9)	14(27.4)	-		10(32.2)		
Chest X Ray(Images)							
Typical	56(94.8)	45(88.2)	1.0 (Reference)	*p* = 0.08	25(80.6)	1.0 (Reference)	*p* = 0.01
Compatible	1(1.9)	6(11.8)	0.13(0.01–1.15)		6(19.4)	0.07(0.008–0.65)	
Atipical	2(3.3)	0	-		0		
Cavitation							
Yes	52(88.1)	40(78.4)	1.0 (Reference)	*p* = 0.16	23(74.2)	1.0 (Reference)	*p* = 0.19
No	6(10.0)	10(19.5)	0.46(0.15–1.37)		7(22.6)	0.37 (0.11–1.25)	
IGN	1(1.9)	1(1.9)			1(3.2)		
Empirical treament							
Yes	58 (98.3)	48(94.1)	1.0 (Reference)	*p* = 0.51	28(90.3)	1.0 (Reference)	*p* = 0.23
1st line drugs	51	34	0.27(0.02–2.73)		12	0.16(0.01–1.61)	
2nd line drugs	7	14			16		
No	1 (1.7)	3 (5.9)			3(9.7)		
Anti_TB drugs prescribed at triage							
Maintained the regimen	18(30.5)	18(35.3)	1.0(Reference)	*p* = 0.59	12(38.7)	1.0 (Reference)	*p* = 0.43
Start new TB regimen	41(69.5)	33(64.7)	1.24(0.56–2.76)		19(61.3)	1.44(0.57–3.57)	
Treatment and Laboratory results among 95 pDR-TB Followed-up							
Adverse reaction							
Yes	7(15.6)	11(22.0)	1.0 (Reference)	*p* = 0.88	8(27.6)	1.0 (Reference)	*p* = 0.82
No	20(44.5)	34(68.0)	0.92(0.30–2.76)		20(68.9)	1.14(0.34–3.75)	
IGN	18(40.0)	5(10.0)			1(3.4)		
Change of treat							
Yes	9(20.0)	27(54.0)	1.0 (Reference)	*p* = 0.03	20(68.9)	1.0 (Reference)	*p* < 0.01
Use DST results	3	21	2.85(1.06–7.60)		16	4.69(1.53–14.34)	
Other	6	6			4		
No	19(42.0)	20(40.0)			9(31.1)		
IGN	17(37.8)	3(6.0)					
AFB 6th month							
Positive	2(4.5)	6(12.0)	1.0 (Reference)	*p* = 0.35	4(13.8)	1.0 (Reference)	*p* = 0.49
Negative	17(37.8)	23(46.0)	2.21(0.39–12.36)		12(41.4)	2.80(0.44–18.0)	
IGN	26(57.8)	21 (42.0)			13 (44.8)		
Culture results 6th month							
Pos	1(2.2)	10(20.0)	1.0 (Reference)	*p* = 0.10	6(20.7)	1.0 (Reference)	*p* = 0.17
Neg	12(26.7)	17(34.0)	7.05(0.79–62.72)		11(37.9)	6.54(0.67–63.33)	
Cont	1(2.2)	1(2.0)	-		0		
IGN	31(68.9)	22(44.0)			12 (41.4)		
Treatment Outcome at 6th month							
Favourable	23(51.0)	33(66.0)	2.42(1.02–5.78)	*p* = 0.04	21(72.4)	2.51(0.92–6.84)	*p* = 0.07
Unfavourable	22(48.9)	13(26.0)	1.0 (Reference)		8(27.6)	1.0 (Reference)	
Default	20	2			1		
Failure	1	10			6		
Death	1	1			1		
Transfer	-	4(8.0)					

*IGN* ignored, *NA* not applicable, *MDR* multidrug resistant, *DR* drug resistant; *DS* drug sensitive

From the 110 pDR-TB included, 95 (86.0%) had been followed-up until the 6th. month. Between those subjects, 45 were DS-TB and 50 were DR/MDR-TB. Among those 95 patients, 24 (25.3%: IC:17.5%–34.9%) changed or initiated the treatment after DST results, totaling 16/29 (MDRTB), 5/21 (DR-TB), and 3/45 (DS-TB cases).

Comparing the treatment outcome in 95 pDR-TB cases followed-up to DS-TB cases, DR/MDR and MDR-TB had higher proportion, respectively, of changing or initiate treatment after DST results (*p* = 0.05, *p* = 0.01) and favourable outcome (*p* = 0.04 and *p* = 0.07) (Table 5).

Among all subjects followed-up, unfavorable outcome was identified in 35 (36.8%; IC: 27.8–46.9), as follows: 2 (2.1%) died, 22 (23.0%) defaulted, 11 (11.6%) failed, and 4 (4.2%) were transferred to another health unit (Table 4). The default rate was higher among DS-TB cases (20/45: 44.4%) than among DR/MDR-TB cases (2/46: 4.3%).

## Discussion

This is a descriptive study, in a high burden country, of the health system approach at reference center level to the investigation and management of patients suspected of having drug resistant TB. At triage in those centers, we observed a high rate (96.0%) of empirical treatment, similar to that reported (96.0%) by Theron et al. [13] among presumed drug sensitive and drug resistant pulmonary TB cases detected by Xpert and higher than that (59.0%) described by Yacobson et al. evaluating presumed drug resistant TB cases [15].

Probably, physicians started anti-TB medication for suspected drug resistant TB before the results of susceptibility testing, due to previous delays experienced in the release of such results by phenotypic tests. Nevertheless, the median release time of DST results was 69.5 days, similar to (52–70 days) observed by Hannarah [10], Tukvadze et al. [11] and Yadava et al. [14] and lower than described (106–133 days) by Boheme et al. [12], Shin et al. [24], and Gler et al. [25].

In our sample, the release median time for the results of susceptibility testing was similar with solid medium (70 days), such as that observed with liquid medium (67 days), which in turn, is different to those results described by Tukvadze et al. [11], where the average time was lower (21.6 days) with liquid medium.

The median time from first admission to the Reference Unit to start the appropriate treatment for DR-TB suspects was 1.0 day, which is lower than that described (67–133 days) by Hannarah et al. [10], Jacobson et al. [15], Joh et al. [26], Shin [24] and Gler [25]. This result may be different as the other studies did not describe the relationship with empirical treatment and the change of treatment after DST results were released.

We observed a large time variation from DST results availability and the start of the appropriate treatment within the 5 reference centers. Major delays in the DST results released by laboratories and seen by physicians occurred in the sites that lacked DST in local laboratories and had no computerized system to release the information to the health care team right away. This confirms that the organization of services should be taken into account when evaluating the incorporation of new diagnostic technologies for TB as commented on by Creswel et al. [16] when implementing Xpert MTB Rif in 9 countries and by Jacobson and Yannarah on the implementation of MDRTB plus in South Africa [10, 15].

High proportions of drug resistant TB (46.0%) and multidrug-resistant TB (25.5%) were found, even in those not previously treated. This results were similar to those described in other studies in which patients with suspected drug resistant TB were evaluated [3, 24, 27, 28]. Among the *M. tuberculosis* strains resistant to rifampicin, 96.0% were resistant to INH, such as described by Kurbatova [29] suggesting that rifampicin resistance can be used as a proxy for MDR-TB.

The factors associated with the occurrence of MDR-TB observed in our study (lower sweating, presence of comorbidities and typical chest x ray images) were similar to those described by Martinez [30]. However, we observed no association of MDR-TB with other variables identified in other studies, such as TB in the past, contact with TB, cavitation in chest X-ray, alcoholism, smoking, type 2 diabetes mellitus, HIV infection, and prison inmates [28, 30–34]. Such results may reflect the small sample size and low frequency of these variables in MDR-TB cases.

In our study, DR and MDR TB cases had higher proportions changing or initiating treatment after DST results and higher favorable outcomes, similar to those described (44.0%–57.0%) by Yacobson [15] and Gler [25].

Compared to DS-TB cases, no difference was observed in culture conversion at the sixtieth month in patients with drug resistant TB, which is a different result from that described by others [10, 15, 30]. At the sixtieth month of follow-up, it was observed a high defaulting rate (23.0%) similar to that described (26.0%) by Tockzek et al. [35] when evaluating 10 studies where no direct observed therapy was performed.

The strengths of our study include: (a) standardized screening of presumed DR TB patients enrolled from 5 Reference Sites in four States; (b) the culture and DST were done in a reference laboratory that follows the standard WHO guidelines and, (c) the personnel performing the DST were unaware of the patient’s clinical or radiographic findings. The limitations of this study are that it relies on small sample size from metropolitan areas within four States, which may not be representative of the whole country, and that we included only patients with culture confirmed TB. Additionally, the exclusion of individuals at high risk for MDR TB that doctors used a previous DST result to manage treatment, may explain the lack of association with factors such as previous TB, contact with TB, cavitation in chest X-ray, alcoholism, smoking, type II diabetes mellitus, as described in the literature.

## Conclusions

This study shows a high rate of empirical treatment and long delays for DST results. Favorable treatment outcomes among DR and MDR-TB patients was due to the adoption of an appropriate treatment, mainly among those that started first line regimens empirically. Improvements in the flow of patients and/or clinical samples to referral centers and, use of triage clinical procedures at referral level with higher performance, may be helpful to provide a more appropriate case management.

## Abbreviations

CI: Confidence interval
CRPHF-Fiocruz-RJ: Centro de Referência Hélio Fraga – Fundação Oswaldo Cruz – Rio de Janeiro
DRTB: Drug resistant tuberculosis
DST: Drug susceptibility test
DSTB: Drug sensitive tuberculosis
EMB: Ethambutol
HM-SES-CE: Hospital Messejana – Secretaria Estadual do Ceará
HU-SES-RGS: Hospital Sanatorio Partenon-Secretaria Estadual de Saúde do Rio Grande do Sul
ICF-SES-SP: Instituto Clemente Ferreira – Secretaria Estadual de São Paulo
IETAP-SES-RJ: Instituto Estadual Ary Parreiras – Secretaria Estadual do Rio de Janeiro
INH: Isoniazid
IQR: Interquartile
LJ: Lowenstein Jensen
MDRTB: Multi-drug resistant tuberculosis
pDRTB: Presumtive diagnosis of drug-resistant tuverculosis
PM: Methods of proportions
PZA: Pyrazinamide
RIF: Rifampicin
TB: Tuberculosis
